# Need for Cognition Is Positively Related to Promotion Focus and Negatively Related to Prevention Focus

**DOI:** 10.3389/fpsyg.2021.606847

**Published:** 2021-07-15

**Authors:** Ashley H. Oiknine, Kimberly A. Pollard, Peter Khooshabeh, Benjamin T. Files

**Affiliations:** ^1^DCS Corporation, Alexandria, VA, United States; ^2^DEVCOM Army Research Laboratory, Los Angeles, CA, United States

**Keywords:** regulatory focus, need for cognition, motivation, five factor personality, behavioral inhibition/behavioral activation systems

## Abstract

Need for cognition (NFC) and regulatory focus (RF) are important variables with individual differences relevant to motivation and goal pursuit. These constructs are widely used in the literature, often separately; no work has simultaneously examined the need for cognition scale (NCS) and Lockwood’s general regulatory focus measure (GRFM). Here, we explore shared theoretical underpinnings of the two constructs and assess whether they may be driven by common underlying factors. Considering purported overlaps between these scales and other constructs (e.g., personality and cognitive processes), we take a strong inference approach to test hypothesized bridges between the two measures. In a large (*N* = 853) sample, we found NCS to be related positively to GRFM promotion and negatively to GRFM prevention scores, suggesting mutual ties with behavioral inhibition system/behavioral activation system, intrinsic motivation, openness, and creativity. A generalized approach motivation, as well as intrinsic motivation, may thus drive both NFC and RF.

## Introduction

Need for cognition (NFC) and regulatory focus (RF) are widely used constructs in the individual difference and motivation literature, often measured using the need for cognition scale (NCS; Cacioppo et al., 1984) and the generalized regulatory focus measure (GRFM; Lockwood et al., 2002), respectively. Here, we identify other dispositional constructs that have shared features and reported commonalities with NFC/NCS and/or RF/GRFM. We then derive hypotheses about the conceptual relationship between NFC and RF. Comparing NCS and GRFM can help determine whether both individual disposition assessments are measuring similar latent variables, how they might differ and can guide other research toward a more contextualized application of their measurement by highlighting these commonalities and differences. Some research has investigated NCS with established individual dispositional traits, such as Higgins’s regulatory focus (Yen et al., 2009). To our knowledge, none have investigated the relationship between NCS and the GRFM. Here, we review some characteristics and theoretical considerations of NFC and RF. Next, we outline evidence leading to various hypotheses about the underlying drivers and overlaps between NFC, RF, and other constructs. Sets of hypotheses are grouped according to what numerical relationship they predict between key measures of NFC and RF (NCS and the subscales of the GRFM, respectively). A strong inference approach is then used to refute or lend support to the differing conceptual hypotheses.

The dispositional trait NFC is characterized as an individual’s tendency to engage in and enjoy effortful thought (Cacioppo and Petty, 1982). Some individuals have a strong tendency to engage in and enjoy effortful thought, while other individuals have a weaker tendency to do this. NFC has been investigated in studies of framing (Chatterjee et al., 2000; Kuvaas and Kaufmann, 2004), motivation (Gottfried et al., 2017), and training (Towler and Dipboye, 2006), among others. These areas of research have benefited from including individual difference traits, such as NFC to uncover moderation effects (Chatterjee et al., 2000).

Many well-studied phenomena impact individuals differently based on their NFC; examples include social loafing (Donohew et al., 2015), framing bias (Tversky and Kahneman, 1981), and the primacy effect (Ahlering and Parker, 1989). This highlights a key difference between those high in NFC (cognizers) and low in NFC (cognitive misers; Taylor, 1981) in their tendencies to avoid or engage in biases/fallacies. Given the NFC’s importance, it is valuable to understand its relationship with other similar but different constructs, such as RF.

RF is a goal orientation construct that describes two motivational foci – promotion and prevention. Promotion focus describes a person’s tendency to pursue gains, whereas prevention focus describes a person’s tendency to avoid loss. These tendencies can also inform strategic inclinations, such as eagerness and avoidance strategies, as well as pursuits of gain or non-loss goal states. Trait regulatory focus is a dispositional variable, and individuals vary in the strength of promotion relative to prevention score. One of the most prominent trait RF measures is the GRFM (Lockwood et al., 2002; Gorman et al., 2012), which allows promotion and prevention aspects of regulatory focus to be measured on separate subscales. The term *reference-point* is used to distinguish between the conceptualizations of Lockwood et al. (2002) relative to others (Summerville and Roese, 2008). The reference-point conceptualization states that prevention focused individuals avoid all possible loss end-states or pursue the absence of a loss state, while promotion focused individuals would pursue a gain end-state or avoid the absence of a gain (Summerville and Roese, 2008). Many researchers use RF to learn about motivation and goal orientation, such as strategic inclinations and other motivational behaviors. This includes investigations of goal framing (Van-Dijk and Kluger, 2004; Vaughn et al., 2008), leadership (Kark et al., 2018), and motivation to attend lectures (Bassili, 2006).

### Hypotheses

Although NFC and RF have largely been treated as independent constructs, their relationship has not been previously studied. Some evidence, reviewed below, suggests that these two constructs might be conceptually related, particularly *via* sharing some common underlying drivers. It is important to note that although we propose shared relations across constructs, we are not necessarily claiming causal relations but rather formulating theory toward understanding how some relations may underlie or help conceptually explain a relationship between NFC and RF constructs (Rohner and Levinsson, 2020). In the following section, we present hypotheses that lead to numerical predictions about the relationships between NCS and the subscales of GRFM (Table 1). When hypotheses make similar or identical predictions for the directionality of these numerical relationships, we have grouped them by the predictions they make.

**Table 1:** Summary of hypotheses and predictions.

Label	Construct	Predicted correlation between NCS and GRFM prevention	Predicted correlation between NCS and GRFM promotion
1a.	Intrinsic/Extrinsic	−	+
1b.	BIS/BAS	−	+
1c.	Openness	−	+
1d.	Creativity	−	+
2.	Elaboration	+	+
3a.	Overthinking/Anxiety	+	
3b.	Conscientiousness	+	
4.	Neuroticism	−	
5.	Easy gains		−

#### Intrinsic Motivation vs. Extrinsic Motivation

Intrinsic motivation in self-determination theory (Deci and Ryan, 1985, 2008) is described as motivation to act rooted in interest and enjoyment. Extrinsic motivation involves the motivation to pursue an external reward (Ryan and Powelson, 1991; Deci and Ryan, 2008). The contrasts between intrinsic and extrinsic motivation are similar to the RF orientations (Van-Dijk and Kluger, 2004).

Previous research has shown positive associations between promotion and intrinsic motivation subscales (i.e., autonomy, competence, and relatedness) and negative associations of these subscales with prevention (Vaughn et al., 2008). The more duty-focused outlook of prevention focused persons comes with a sensitivity to others’ expectations, pursuit of the ought-self (Summerville and Roese, 2008), and sensitivity to external motivation (Lalot, 2018). Promotion focused persons pursue their own inherent goals (Crowe and Higgins, 1997; Brockner and Higgins, 2001) and are not as sensitive to external reward, suggesting a more intrinsically motivated orientation.

NFC describes individual dispositional differences in intrinsic motivation to engage in effortful thought (Cacioppo and Petty, 1982; Petty and Cacioppo, 1986). Research has reported cognizers’ tendency to be relatively intrinsically motivated (Petty and Cacioppo, 1986; Furnham and Thorne, 2013). Although NCS score does not predict success on tasks per se, it does make predictions regarding an individual’s tendency to engage in effortful thinking tasks. Those with a low NCS score perceive thinking as a chore that is reluctantly engaged in when some extrinsic incentive is involved (Thompson et al., 1993; Petty et al., 2009).

NCS score and intrinsic motivation have been shown to be positively correlated. Intrinsic motivation also overlaps with the self-guide conceptualization of RF. This overlap points us to the hypothesis:

H1a: Individual differences in intrinsic motivation drive an individual’s tendency to engage in effortful and goal-oriented thinking, while individual differences in extrinsic motivation drive an individual’s tendency to engage in duty-focused thinking.

Under this hypothesis, we predict a positive relationship between promotion focus score and NCS score as well as a negative relationship between prevention score and NCS score. However, there are also other hypotheses that make the same numerical prediction.

#### Behavioral Inhibition System/Behavioral Activation System

The biopsychological theory of personality (Gray, 1982) includes the behavioral inhibition system (BIS) and behavioral activation system (BAS). BIS is related to the avoidance of adverse outcomes, and BAS is related to approach motives toward goals. The BIS factor is sensitive to signals of punishment, anxiety, harm avoidance, and non-reward, whereas the BAS factor is sensitive to positive outcomes, positive affect, fun-seeking, novelty, and non-punishment (Carver and White, 1994; Haws et al., 2010). Fundamentally, the dispositional construct BIS/BAS is about individual differences in goal orientation and motivation, similar to GRFM and NCS.

Prevention focused individuals are described as sensitive to punishment, anxious, and avoidant of loss (Higgins, 1998; Lockwood et al., 2002; Fleischhauer et al., 2010). These descriptions have overlap with characteristics and expressions of BIS. Similarly, approach characteristics overlap with gain orientation of promotion focus. Both promotion and BAS involve goal orientation and positive affect (Fleischhauer et al., 2010; Gorman et al., 2012). GRFM promotion scores have a strong positive relationship with BAS, as do GRFM prevention scores with BIS (Summerville and Roese, 2008; Haws et al., 2010). BIS/BAS has sometimes been recommended and explicitly used as a proxy measure of RF (Haws et al., 2010; Gorman et al., 2012).

NFC has been associated with approach and elements of BAS, such as novelty and enjoyment of complex tasks (Cacioppo and Petty, 1982; Carver and White, 1994; Nussbaum and Bendixen, 2003). NCS has shown negative relationships with avoidance properties, such as harm avoidance (Fleischhauer et al., 2010). Moreover, NCS has been shown to relate positively to BAS and negatively to BIS (Gray, 1982; Fleischhauer et al., 2010). This would lead to the following hypothesis:

H1b: Individual differences in approach and avoidance tendencies motivate a person toward or away from effortful thinking and toward promotion oriented or prevention oriented thinking, respectively.

Under this hypothesis, we would predict that NCS score would positively relate to promotion score and negatively to prevention score.

#### Openness

Openness is one factor within the big five personality inventory (McCrae and Costa, 1992). Individuals high in openness are characterized as culturally sophisticated, imaginative, curious, creative, sensitive to emotion, novelty seeking, intellectual, reflective, and thoughtful about ideas (McCrae and Costa, 1997).

Openness has been shown to be positively associated with promotion-related goals (Vaughn et al., 2008). Some work has suggested that promotion and openness are so strongly related that openness is sometimes used as a proxy for promotion (Bassili, 2006). Whereas prevention focused persons are more concerned with behaving in a way that is safe and not risky, suggesting a negative relationship with openness, promotion focused persons are more concerned with approaching gains and are more inclined to be open to experience regardless of risks (Friedman and Förster, 2001).

People with a high NFC tend to enjoy engaging in complex problems (Nair and Ramnarayan, 2000), similar to open individuals’ tendency to be curious and their attraction to ideas (Mussel, 2010) and novel experiences. Those high in NFC more strongly tend to elaborate and evaluate incoming information (Petty and Cacioppo, 1986) as do those who are high in openness (McCrae and Costa, 1997). Cognizers and those high in openness pursue novel experiences (Olson et al., 1984). Much work shows openness and NCS have a strong positive relationship (Tuten and Bosnjak, 2001; Fleischhauer et al., 2010; Mussel, 2010; Furnham and Thorne, 2013; Lins de Holanda Coelho et al., 2018). This brings us to the hypothesis:

H1c: The personality trait of openness motivates an attraction to novel, complex problems, and goals without regard to risk-taking, while lack of openness motivates an aversion to risk.

Under this hypothesis, we would predict that NCS score would have a positive relationship with promotion score and negative relationship with prevention score.

#### Creative and Exploratory Processing

The motivational aspects of creativity, such as risk taking and desire for novelty retain much overlap with RF and NFC.

There is some evidence that suggests that promotion orientation may be conducive to creativity (Kark et al., 2018), potentially *via* cognitive tuning theory. Cognitive tuning theory (Schwarz, 1990) suggests that positive affective environments encourage exploratory behavior and support creative thought (Friedman and Förster, 2001; Zhu and Meyers-Levy, 2007; Bonetto et al., 2020). The literature has reported strong associations between promotion focus and positive affective states (Summerville and Roese, 2008), and positive affect and creativity (Friedman and Förster, 2001, 2002). Some literature has reported promotion focused persons display superior performance on tasks involving creativity compared to prevention focused persons (Crowe and Higgins, 1997; Friedman and Förster, 2001).

RF and NFC may share aspects related to creativity that may explain their relationship to each other. Much literature shows differences in creative ability of cognizers as compared to cognitive misers (Chen et al., 2006; Dollinger, 2011; Watts et al., 2017). On a conceptual level, NFC and creativity share many facets, namely, tolerance of ambiguity, a propensity to approach novelty, and comfort with complexity (Petty and Cacioppo, 1986). This conceptual overlap has also surfaced in the literature. For example, one researcher investigated creative outcomes of individuals varying in levels of NCS and reported that cognizers displayed more creative past accomplishments, more individualistic photograph essays, and significant associations with the Hocevar’s creative behavior inventory (Dollinger, 2011). This leads us to the hypothesis:

H1d: Positive affect and exploratory behavior are hallmarks of high NFC and conducive to creativity, while intolerance for ambiguity and risk (i.e., prevention focus) are not.

Under this hypothesis, we predict a positive relationship between promotion score and NCS score as well as a negative relationship between prevention score and NCS score.

In summary, these hypotheses (H1a–d; Table 1) predict a positive relationship between NFC and promotion and a negative relationship between NFC and prevention based on the previous literature involving individual differences in (1) intrinsic motivation, (2) approach-avoidance systems, (3) openness factor of personality, and (4) creativity. Note that none of these hypotheses are necessarily mutually exclusive, and indeed, they all predict the same relationship. Should this relationship surface, teasing apart these individual hypotheses may serve as a starting point to future work more precisely modeling the relationship between RF and NFC.

#### Elaboration

Elaboration is the extent to which a person is motivated to engage in issue-relevant thinking and to scrutinize incoming information (Petty and Cacioppo, 1986).

Research has shown that both promotion and prevention focused individuals engage in elaboration (Zhu and Meyers-Levy, 2007; Choi et al., 2017). Prevention focused persons tend to engage in item-specific elaboration (i.e., context specific associations), while promotion focused persons tend to engage in relational elaboration (i.e., associations between abstractions). Although these are different elaboration styles, it lends support to the claim that both subscales of RF include an elaboration component.

One major feature of NFC involves elaborative thinking; those low in NFC elaborate on incoming information less than those with a high NFC (Petty and Cacioppo, 1986). For example, individuals’ varied responses to framing can be driven by high NFCs’ tendency to elaborate. In Smith and Levin’s investigation of framing effects and NFC, they presented participants with the “ticket problem,” framed in different ways (Tversky and Kahneman, 1981). Results indicated that low NFC persons varied their responses based on the framing of the problem, a characteristic of low elaboration, while high NFC persons gave consistent responses independent of framing – indicative of the elevated elaborative tendencies in high NFC participants (Smith and Levin, 1996).

In summary, high elaborative tendencies are observed in persons high in GRFM prevention and promotion (relational and item specific) and in persons high in NFC. This brings us to the hypothesis:

H2: Individual differences in enjoyment of complex problems, pursuit of gains, and loss avoidance differently motivate the elaboration and scrutiny of incoming information.

Under this hypothesis, we would predict a positive relationship between both GRFM subscale scores and NCS score.

#### Elaboration to the Point of Overthinking

Overthinking, or excessive rumination, describes a state in which one is extensively elaborating in a self-reflective context (Smith and Alloy, 2009). Overthinking is important to understand for motivation, especially when perceived as adaptive for goal attainment (e.g., planning).

Prevention focused persons’ risk-averse outlook involves evaluating potential risks in the motivation to avoid them. It is clear how evaluating risks can leave the prevention focused person tempted to overthink. Prevention focus has been shown to be related to anxiety (Klenk et al., 2011) and negative affect (Summerville and Roese, 2008). Similar to prevention, higher rumination was reported to be associated with negative mood (Genet and Siemer, 2012). There is evidence that prevention focused persons with prevention goal regrets had a tendency to ruminate on alternative decisions (Leder et al., 2013).

Given cognizers’ tendency to elaborate, cognizers may also be susceptible to overthinking. Which brings us to the hypothesis:

H3a: Elaborate scrutiny of complex problems and risk-avoidant thinking are conducive to overthinking and rumination.

Under this hypothesis, we might predict a positive association between NCS score and prevention score.

#### Conscientiousness

Conscientiousness is another big five personality trait of likely relevance. Conscientious persons are efficient, organized, careful, thorough, and disciplined (McCrae and Costa, 1992; John and Srivastava, 1999), which is especially valuable for goal-related behavior.

Prevention focus involves planning and avoiding risk, which may require thoughtfulness and detail orientation. Prevention focus also involves high levels of risk aversion, dutifulness, vigilance, and conscientious self-regulation (Brockner and Higgins, 2001). Themes of vigilance, dutifulness, and impulse control occur in both conscientiousness and prevention constructs (for example, Crowe and Higgins, 1997; Rose et al., 2002).

NFC has elements that overlap with and are positively related to conscientiousness (Tuten and Bosnjak, 2001). Research has shown that achievement striving (sub-factor of conscientiousness) and NFC have a strong positive relationship (*r* = 0.44; Fleischhauer et al., 2010). Much literature shows strong relationships between overall conscientiousness and NFC with correlations ranging from 0.20 to 0.40 (Bye and Pushkar, 2009; Dollinger, 2011; Furnham and Thorne, 2013).

Given the significant overlap between conscientiousness and NFC and prevention, it is reasonable to hypothesize that conscientiousness may underlie both prevention focus and NFC. No straightforward hypothesis for promotion and NFC can be posited based on the reviewed conscientiousness literature. Promotion focused people are inherently risk seeking, and the promotion scale has not been shown to include prominent features of conscientiousness.

H3b: Conscientiousness encourages achievement striving and vigilant behavior.

Under this hypothesis, we would predict a positive association between NCS score and prevention score.

In summary, the aforementioned hypotheses (H3a–b; See Table 1) predict a positive relationship between NFC and prevention based on conscientiousness or the tendency to overthink. Should this relationship surface, future work can more deeply investigate the relative contribution of these factors.

#### Neuroticism

Neuroticism (a big five personality trait) describes individuals who are often anxious, worried, vulnerable to stress, and fearful (John and Srivastava, 1999; Schneider, 2004; Paulus et al., 2016). Individuals who are low in neuroticism can be described as calm and stable while high neuroticism involves negative affectivity and emotionality (John and Srivastava, 1999). Since affect has implications for motivation, neuroticism is an important facet of personality to pay attention to when understanding motivation and self-regulation. People who are low in neuroticism have higher rationality, stability, and consistency compared to their high neuroticism counterparts (McCrae et al., 1986).

Summerville and Roese report that GRFM prevention scores were related to negative affect (Summerville and Roese, 2008). Additionally, research has acknowledged a close link between properties of prevention and neuroticism (Keller et al., 2008) and has used neuroticism as a proxy measure for prevention focus (Bassili, 2006).

The negative relationship between NFC and neuroticism is well established (Sadowski and Cogburn, 1997; Dollinger, 2011; Furnham and Thorne, 2013), but see Woo et al. (2007) who found only a small negative relationship. This largely negative relationship may be attributable to neuroticism’s sensitivity to anxiety (McCrae et al., 1986; Jorm et al., 2000; Paulus et al., 2016). NFC has a negative relationship with anxiety (Olson et al., 1984) which may partially explain its relationship with neuroticism. This points us to the hypothesis:

H4: Neuroticism motivates negative affective states while the enjoyment of elaborative thought does not.

Under this hypothesis, we would predict prevention score and NCS score would have a negative relationship.

#### Easy Gains and Complex Losses

High NFC persons have a tendency to prefer complex tasks, and this may suggest that high NFC persons see easy tasks (those requiring little effortful thought to complete) as perhaps trivial and not worth pursuing. Although some literature suggests that high NFC persons do not dismiss easy tasks (Fisher and Oyserman, 2017), their preference for more complex tasks may interfere with their ability to amass a series of gains from easy tasks. This would lead to the hypothesis:

H5: The pursuit of easy gains and pursuit of complex tasks are opposing motivations.

Under this hypothesis, we might predict a negative relationship between NCS score and promotion score. Unfortunately, little work has been done on promotion focused persons’ tendency to pursue easy compared to difficult tasks, making this prediction quite speculative but still plausible. If this relationship was to surface, more work would be needed to establish these discrete task/gain value systems and the degree to which they drive the conceptual relationship between RF and NFC.

In summary (Table 1), the literature suggests several hypotheses that conceptually link the dispositional traits of NFC and regulatory focus. These hypotheses predict different numerical relationships between key measures: NCS and the two subscales of the GRFM. We employ a strong inference approach using a large sample of participants to help determine which of these hypotheses best explain the relationship of these two motivational constructs. The true relationship between these dispositional constructs is likely complex and dependent on many underlying factors with varying effect sizes. The goal of the following analysis is to disconfirm a subset of these hypotheses as describing a major connection between NFC and RF.

## Materials and Methods

The methods and hypotheses for this study were not pre-registered. Data and code for analyses are available online (Oiknine, 2019). These data were collected as a part of an overarching project containing various studies (Files et al., 2019). We have reported how we determined our sample size, all data exclusions, all manipulations, and all measures in the study.

### Participants

A total of 1,398 participants completed a web-based questionnaire. 853 (586 F, 265 M, 2 chose not to specify gender) participants completed all items; all incomplete participants’ data were omitted from current analyses. The participants’ ages ranged from 18 to 65 years with a mean of 21 years. Our sample size for this study was not predetermined. Our study was advertised for 6 months until a parent study (Files et al., 2019) was complete. We took data from all participants who responded to our ad during this time window. All participants were recruited from online platforms associated with the community of the University of California, Santa Barbara (e.g., SONA). The voluntary consent of participants in this research was obtained as required by Title 32, Part 219 of the CFR, and Army Regulation 70–25. All human subjects testing was approved by the Institutional Review Board of the United States Army Research Laboratory (protocol number 17–017) as well as the Institutional Review Board at the University of California, Santa Barbara.

### Procedure

Participants used their own electronic devices (e.g., laptops, computers, and mobile phones) to complete the survey implemented with the Qualtrics software. After a consent document and overview of the questionnaire, participants answered demographic questions (e.g., age and gender), a modified version of GRFM (Lockwood et al., 2002), and NCS (Cacioppo and Petty, 1982). Time to complete the questionnaire was estimated at ~10 min.

### Measures

We collected only NCS, GFRM, and demographic information. Demographic information was not used in the analyses except to describe our sample.

A modified version of Lockwood’s GRFM was used to measure relative prevention and promotion strength. We did not present items (items 7, 8, 12, and 13) in the Lockwood survey that pertained to academic or school involvement (e.g., “My major goal in school right now is to avoid becoming an academic failure.”) to ensure the questions were equally relatable and relevant to all participants (Haws et al., 2010). This measure contained 14 items that were rated using a 9-point scale with anchors at “1 not at all true of me” to “9 very true of me.” Lockwood reported reliability for the promotion and prevention subscales as *α* = 0.81 and *α* = 0.75, respectively. However, in a meta-analysis of 30 administrations of the GRFM, the average Cronbach’s alpha was *α* = 0.82 for both subscales (Gorman et al., 2012). The GRFM prevention and promotion subscales have a small but significant association (*r* = 0.17, *p* < 0.01; Lockwood et al., 2002).

Participants completed the 18 item NCS (Cacioppo and Petty, 1982; Cacioppo et al., 1984). NCS was rated along a 5-point Likert scale with anchors at “1 extremely uncharacteristic of me,” “2 somewhat uncharacteristic of me,” “3 uncertain,” “4 somewhat characteristic of me,” and “5 extremely characteristic of me.” The reported alpha for the scale is 0.90 (Cacioppo et al., 1984).

### Analyses

The scoring for the NCS was in accordance with Cacioppo et al. (1984). A subset of the NCS was reverse scored (for items 3, 4, 5, 6, 7, 8, 12, 16, and 17), and values were summed across all 18 items. The GRFM promotion score and prevention scores were calculated using the guidance outlined in Lockwood et al. (2002). Promotion and prevention scores were calculated by computing the average across all subscale items (two separate scores).

Pearson correlations were then computed between NCS and promotion score, and between NCS and prevention score. A non-parametric bootstrapped CI approach was included to evaluate the relationship between each of the variables.

## Results

**Figure 1:**
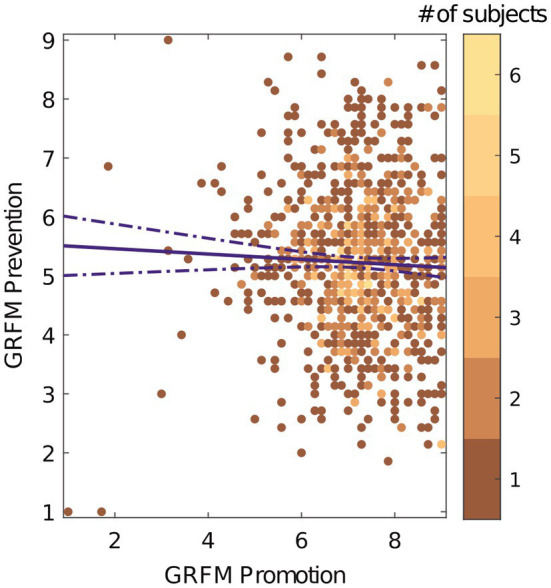
GRFM promotion and prevention subscales are un-correlated. The color bar represents number of subjects. The solid purple line indicates the direction of the relationship between the two scales. The dashed line is the 95% confidence interval.

We computed the internal consistency using the alpha coefficient for the altered GRFM questionnaire used to see if our modifications changed the value. Both promotion and prevention scales displayed high alpha coefficients (promotion, *α* = 0.82; bias-corrected accelerated bootstrapped 95% *CI* = [0.80, 0.85]; and prevention, *α* = 0.71, 95% *CI* = [0.68, 0.75]). The two subscales did not have a significant relationship (*r* (851) = −0.038, 95% *CI* = [−0.118, 0.072], *p* = 0.262; see Figure 1). Our reported confidence interval for promotion contains both the reported alpha values from Lockwood et al. (2002; *α* = 0.81, *α* = 0.75) and Gorman et al. (2012; *α* = 0.82, *α* = 0.82). The alpha for the prevention subscale was marginally lower than that reported by Lockwood and well below that in Gorman’s meta-analysis.

**Figure 2:**
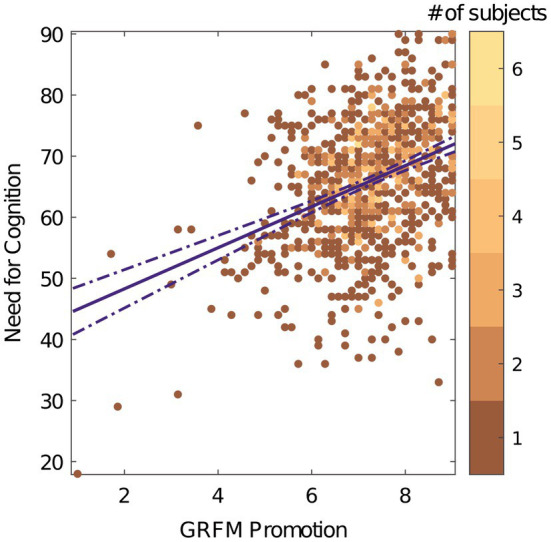
GRFM promotion vs. NCS. The color bar represents number of subjects. The solid purple line indicates the direction of the relationship between the two scales. The dashed line is the 95% confidence interval.

**Figure 3:**
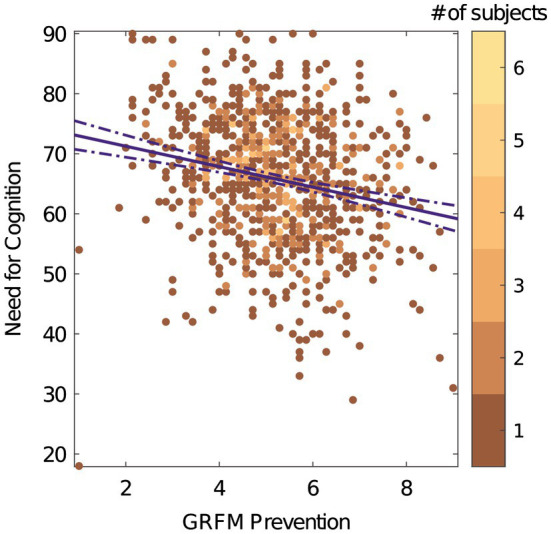
GRFM prevention vs. NCS. The color bar represents number of subjects. The solid purple line indicates the direction of the relationship between the two scales. The dashed line is the 95% confidence interval.

The promotion subscale showed a positive correlation with NCS (*r* (851) = 0.36, *p* < 0.001, 95% *CI* = [0.289, 0.435]; Figure 2). The prevention subscale showed a negative correlation with NCS (*r* (851) = −0.21, *p* < 0.001 95% *CI* = [−0.281,−0.120]; Figure 3). These results are consistent with the directional predictions made by hypotheses 1a–d and 4 and are inconsistent with hypotheses 2, 3a, 3b, and 5.

## Discussion

NCS was positively related to promotion score and negatively related to prevention score, consistent with the predictions made by hypotheses 1a–1d and 4 (see Table 1). These results did not support hypotheses 2, 3a, 3b, and 5.

Rejected hypotheses include H2 (elaboration), H3a (overthinking), H3b (conscientiousness), and H5 (easy gains). Because prevention and promotion focus are associated with elaboration, as is NFC, a tendency to elaborate – and perhaps a latent individual trait construct having to do with elaborative tendencies – was hypothesized to underlie both NFC and RF. If this was the case, we would predict that NCS would be positively related to both prevention and promotion. However, while NCS was found to be positively related to promotion score in our sample, it was not positively related to prevention score. This may mean that elaborative tendencies are not a major driving force of both RF and NFC. It also may mean that elaborative tendencies are perhaps only a driving force behind promotion. As promotion-focused and prevention-focused persons perform their elaboration in very different ways (Zhu and Meyers-Levy, 2007; Choi et al., 2017), it remains possible that a more nuanced understanding of elaborative tendencies might have predictive value for GRFM. Future research can aim to develop tools to assess these tendencies and to explore their relationship to other motivation-related individual traits.

H3a, the idea that highly prevention-focused persons might be prone to overthinking, and that overthinking may also result from high NFC, leads to the prediction that NCS and prevention scores might be positively related. Our results suggest the opposite. It is possible that overthinking is not more common with higher prevention scores, and possible that high NFC does not encourage more overthinking, perhaps because NCS is associated with more positive affect.

H3b postulated that the big five personality factor of conscientiousness might be a major driver underlying both prevention focus and NFC. Conscientiousness includes aspects, such as organization, vigilance, dutifulness, carefulness, and impulse control. Our results indicate that, contrary to the predictions made by the conscientiousness hypothesis, NCS and prevention score are negatively, not positively, related. This may be due to incomplete overlap between aspects of conscientiousness and aspects of prevention focus and NFC. It may be that prevention focus is not as much about organization and efficiency as conscientiousness is. While prevention focused people may be more vigilant and risk-averse, it is possible that prevention focus does not have much to do with detail orientation and order but rather the duty and obligation surrounding it. NFC, on the other hand, may largely relate to achievement striving, which is an aspect of conscientiousness unlikely to correlate with prevention focus. It may also be the case that conscientiousness is a driver of both NFC and prevention, but that other latent traits (such as openness or BIS/BAS orientation) exert a larger effect. Future studies can employ detailed modeling to examine this possibility.

Regarding H5, effortful thinking is not necessarily required in order to achieve gains. Therefore, a high NFC person, who may find easy tasks not worth pursuing, may miss out on easy gains that a high promotion person might value. To the extent that this is a common occurrence, we would predict NFC to be negatively related to promotion (H5). Our data found the opposite relationship, suggesting that this is not a particularly common occurrence or that other factors are strong enough to override any such effects. Little research has been done on RF and NFC and the pursuit of easy vs. difficult gains, so this remains an open topic for further examination. Future work would benefit from investigating differences in how promotion focused and high NFC persons perceive difficult tasks and gains as well as their importance or value appraisals in relation to their difficulty. High NFC participants may enjoy complex tasks especially when there are gains involved and low NFC participants may avoid complex tasks regardless of aspirations and gains.

Supported hypotheses were H1a–d and H4. H1 predicted that NCS is positively related to promotion score and negatively related to prevention score, a relationship that could be predicated on a mutual relationship with locus of motivation (intrinsic vs. extrinsic, H1a), general approach/inhibition orientation (BIS/BAS, H1b), openness to experience (H1c), and/or creativity (H1d). In thinking about how constructs in H1 may be interrelated, it is interesting that the previous literature has often measured many of these constructs together. For example, one study aimed at validating a measure of autonomy used approach/avoidance, intrinsic motivation, RF, and openness for construct validity (Cooper et al., 2015), suggesting a commonality across these constructs. Of our four hypotheses, heightened creativity was hypothesized as a potentially shared result or expression of both NFC and RF. Some literature has shown positive relationships between openness and creativity moderated by motivation (Sung and Choi, 2009). It may be that the underlying driver(s) of NFC and RF may also be expressed in creativity. Overall, the hypothesized drivers seem to ultimately reduce down to approach and avoidance behavior. Intrinsic motivation involves an internal desire to approach some task, which is the same for approach in BIS/BAS. The connection between BAS and openness (Fleischhauer et al., 2010) also has to do with approaching instead of avoiding (e.g., novel ideas, experiences, and perspectives). Unfortunately, our current data do not allow us to tease apart the relative contributions of these different potential underlying factors; however, the overall relationship suggests that one or more of these factors may be a significant driver of both NFC and RF, or indeed, that an underlying approach/avoidance tendency may influence NFC and RF as well as locus of motivation, openness to experience, and creativity.

The negative relationship between NFC and prevention could additionally be explained by underlying levels of the five factor trait neuroticism (H4). H4 hypothesized that neuroticism might underlie both prevention focus and NFC. While it is straightforward to suspect that anxiety-prone attributes of neuroticism may lead a person to have elevated focus on avoiding losses, it is less clear why it would lead a person to prefer to avoid effortful thought. However, effortful tasks, including effortful thinking, inherently carry some risks of failure. It is possible that an anxious person may be more comfortable avoiding these effortful and risky tasks, whether in the realm of effortful thinking or in other risky situations, thus leading to a negative relationship between prevention and NFC.

While the current work has strengths that contribute to the motivation and individual differences literature, there are limitations that should be taken into account. Although our respondents covered a wide age range (18–65 years), most were young adults (mean = 21 years). Our population was drawn from a participant pool and other online platforms associated with the community of the University of California, Santa Barbara, so these findings may be primarily applicable to 18–24-year-olds who are pursuing or who have pursued university education. Additionally, we omitted items within the Lockwood measure that we suspected would not be equally applicable to all participants. Our resulting alpha coefficients for the promotion scale agree with values reported by Lockwood et al. (2002) and Gorman et al. (2012). However, the prevention focus reliability we found (*α* = 0.71, 95% *CI* = [0.68, 0.75]) did not agree with what Gorman found (see Table 2). The internal reliability of the prevention subscale of our modified instrument was marginally smaller than the value reported for the full instrument (Lockwood et al., 2002), although the value Lockwood reported was not included within our reported confidence interval. One potential explanation could be the lower number of items in our modified GRFM survey may be contributing to the lower alpha, as alpha is biased upward by increased number of items (Cortina, 1993). Future work should investigate what the potential impact of the items omitted is on the relationship with NFC while incorporating the participant pool biases (e.g., non-students). While we do not have reason to believe that the results depend on characteristics of the participants or context, we do not have any evidence that the findings will occur outside of this population with the modified Lockwood GRFM and the 18 item NCS.

**Table 2:** Reported alpha coefficients.

Citation	Prevention *α*	Promotion *α*
This study	0.71	0.82
Gorman et al., 2012	0.82	0.82
Lockwood et al., 2002	0.75	0.81

The current work took a strong inference approach to evaluate hypotheses about potential conceptual relationships and underlying drivers of NFC and RF. These hypotheses yielded a series of differing predictions regarding the expected mathematical relationship between measurements of these constructs, NCS and GRFM, respectively. After outlining various predictions and literature, we analyzed the strength and direction of the relationship between GRFMs’ subscales and NCS. In doing so, we disconfirmed a subset of hypotheses involving potential major drivers underlying NFC and RF. Results indicate a positive relationship between NCS and promotion score and a negative relationship between NCS and prevention score, consistent with the hypotheses that NFC and RF traits may be driven by underlying personality factors, such as openness to experience, locus of motivation (intrinsic vs. extrinsic), or approach and avoidance tendencies (BIS/BAS), and may be mutually related to creativity. While our approach does not discriminate among these hypotheses, it holds up these factors as being potential candidates for latent underlying individual traits or drivers of both NFC and RF. It should be noted that these candidates are not mutually exclusive and indeed have some common themes. Whether it has to do with gains, complex tasks, or exploring environments, many of these hypotheses could be broadly viewed in terms of approach and avoidance. Future work could employ advanced mediation analyses to more precisely determine whether a particular form of approach/avoidance, or perhaps approach/avoidance in general, underlies both NFC and regulatory focus.

We sought to learn more about whether both assessments (NCS and GRFM) were measuring similar latent variables, to learn how they might differ and in what ways, and to guide other research toward a recommended measurement especially in motivational and goal orientation research domains. We hope that future work will benefit from utilizing these measures with a closer idea of their potential underpinnings in mind and using similar methods to interrogate other widely used measures of interest.

## Data Availability Statement

The data and code used for this study can be found in the Open Sciences Framework in a publicly available repository (doi: 10.17605/OSF.IO/9C4PT).

## Ethics Statement

The studies involving human participants were reviewed and approved by United States Army Research Laboratory Internal Review Board. The participants provided their written informed consent to participate in this study.

## Author Contributions

AO: conceptualization, writing - original draft, resources, investigation, and formal analysis. KP: conceptualization, writing - original draft, resources, and methodology. PK: conceptualization and writing - reviewing and editing. BF: conceptualization, formal analysis, writing - reviewing and editing, and methodology. All authors contributed to the article and approved the submitted version.

### Conflict of Interest

The author AO was employed by the company DCS Corporation.

The remaining authors declare that the research was conducted in the absence of any commercial or financial relationships that could be construed as a potential conflict of interest.
